# Diagnostic yield of genetic testing in a heterogeneous cohort of 1376 HCM patients

**DOI:** 10.1186/s12872-021-01927-5

**Published:** 2021-03-05

**Authors:** Julie Hathaway, Krista Heliö, Inka Saarinen, Jonna Tallila, Eija H. Seppälä, Sari Tuupanen, Hannu Turpeinen, Tiia Kangas-Kontio, Jennifer Schleit, Johanna Tommiska, Ville Kytölä, Miko Valori, Mikko Muona, Johanna Sistonen, Massimiliano Gentile, Pertteli Salmenperä, Samuel Myllykangas, Jussi Paananen, Tero-Pekka Alastalo, Tiina Heliö, Juha Koskenvuo

**Affiliations:** 1 Blueprint Genetics, a Quest Diagnostics Company, 2505 3rd Ave, Suite 204, Seattle, 98121 USA; 2 Heart and Lung Center, Meilahti Tower Hospital, Helsinki University Hospital, Haartmaninkatu 4, P.O. Box 340, 00029 Helsinki, Finland; 3 Blueprint Genetics, a Quest Diagnostics Company, Keilaranta 16 A-B, 02150 Espoo, Finland

**Keywords:** Hypertrophic cardiomyopathy, Genetic testing, Next generation sequencing, Diagnosis, Counseling

## Abstract

**Background:**

Genetic testing in hypertrophic cardiomyopathy (HCM) is a published guideline-based recommendation. The diagnostic yield of genetic testing and corresponding HCM-associated genes have been largely documented by single center studies and carefully selected patient cohorts. Our goal was to evaluate the diagnostic yield of genetic testing in a heterogeneous cohort of patients with a clinical suspicion of HCM, referred for genetic testing from multiple centers around the world.

**Methods:**

A retrospective review of patients with a suspected clinical diagnosis of HCM referred for genetic testing at Blueprint Genetics was undertaken. The analysis included syndromic, myopathic and metabolic etiologies. Genetic test results and variant classifications were extracted from the database. Variants classified as pathogenic (P) or likely pathogenic (LP) were considered diagnostic.

**Results:**

A total of 1376 samples were analyzed. Three hundred and sixty-nine tests were diagnostic (26.8%); 373 P or LP variants were identified. Only one copy number variant was identified. The majority of diagnostic variants involved genes encoding the sarcomere (85.0%) followed by 4.3% of diagnostic variants identified in the RASopathy genes. Two percent of diagnostic variants were in genes associated with a cardiomyopathy other than HCM or an inherited arrhythmia. Clinical variables that increased the likelihood of identifying a diagnostic variant included: an earlier age at diagnosis (*p* < 0.0001), a higher maximum wall thickness (MWT) (*p* < 0.0001), a positive family history (*p* < 0.0001), the absence of hypertension (*p* = 0.0002), and the presence of an implantable cardioverter-defibrillator (ICD) (*p* = 0.0004).

**Conclusion:**

The diagnostic yield of genetic testing in this heterogeneous cohort of patients with a clinical suspicion of HCM is lower than what has been reported in well-characterized patient cohorts. We report the highest yield of diagnostic variants in the RASopathy genes identified in a laboratory cohort of HCM patients to date. The spectrum of genes implicated in this unselected cohort highlights the importance of pre-and post-test counseling when offering genetic testing to the broad HCM population.

**Supplementary Information:**

The online version contains supplementary material available at 10.1186/s12872-021-01927-5.

## Background

Hypertrophic cardiomyopathy (HCM) is an inherited cardiac disorder that is defined by the presence of increased left ventricular (LV) wall thickness that is not solely explained by loading conditions [1]. In terms of genetic conditions, it is relatively common; historical studies have estimated a prevalence of 1 in 500 [2], but recent work demonstrates this to be closer to 1 in 200 [3]. HCM is clinically heterogenous as individuals with severe hypertrophy may remain asymptomatic but those with mild hypertrophy may develop significant arrhythmias, heart failure and/or sudden death. Classic HCM is primarily caused by variants in the genes encoding proteins of the cardiac sarcomere [4] and follows an autosomal dominant pattern of inheritance. Although rarer, a number of multisystem diseases are genocopies of HCM, which require additional surveillance, treatment options, and may follow a different inheritance pattern. Some examples include Fabry disease, *PRKAG2*-related disease, Danon disease, neuromuscular diseases, mitochondrial myopathies and the RASopathies such as Noonan syndrome. Obtaining a correct diagnosis is therefore of utmost importance for medical management purposes, but also for the identification of at-risk family members who require ongoing screening.

Genetic testing provides one way in which a diagnosis can be confirmed in this patient population. Although clinical genetic testing for HCM has been available since 2003 [4], its diagnostic yield in patients with HCM has remained relatively constant, despite advances in testing technology, broader gene inclusion, established reference databases, and guidelines to improve variant interpretation.

Clinical and laboratory cohorts from the pre-next generation sequencing (NGS) era report a yield in the range of 30–40% [5–8] similar to what has been reported in studies utilizing NGS [9–13]. Variant interpretation in most of the older published studies (prior to 2015) did not follow a systematic variant classification scheme such as the ACMG (American College of Medical Genetics and Genomics)—AMP (Association for Molecular Pathology) 2015 guidelines [14]. Integration of copy number variant (CNV) analysis has not significantly increased the yield [11, 15]. The sensitivity of CNV analysis using NGS is constantly improving; it is highly dependent on bioinformatics pipeline and data interpretation. Smaller variants such as exon-level deletions or duplications may not always have been detected [16].

Although NGS has enabled high throughput testing, studies with clinical cohorts have repeatedly shown that increasing the number of analyzed genes does not significantly increase the yield [12, 17]. Recent work by the HCM ClinGen Gene Curation Expert Panel determined that only 46% of evaluated HCM/intrinsic cardiomyopathy and syndromic genes associated with isolated left ventricular hypertrophy (LVH) routinely included on testing panels were categorized as having a definite, strong or moderate association with the disease [18]. Among these are three RASopathy genes (*RAF1, RIT1* and *PTPN11*), which have been shown to cause isolated LVH in some patients, but have only been comprehensively evaluated in a small number of studies [19, 20]. An absence of syndromic features in some patients could be explained by mild syndromic gestalt and/or low to intermediate level mosaicism, which may not be detected by certain NGS platforms without sufficient read depth.

Studies of well-defined clinical cohorts have reported similar diagnostic yields as presumably more heterogeneous referral laboratory cohorts. However, one of the largest HCM laboratory cohorts published to date [9] excluded patients with left ventricular hypertrophy explained by a clinical syndrome. Due to their more stringent inclusion criteria, the reported yield (32%) may not be completely representative of a broad HCM population.

Clinical variables that increase the likelihood of a positive genetic test result include a positive family history [5–7, 9, 21], an earlier age of onset/diagnosis [5–7, 9, 21], maximal left ventricular wall thickness (MLVWT) [6], and specific types of septal morphology [8]. While these factors have been compiled into genotype-predictor scores by two different groups [8, 22], they have been primarily used and validated in well-defined cohorts.

Genetic testing for any patient with HCM has become a guideline-based recommendation [1, 23], suggesting that it may be increasingly performed in patients with a lower index of suspicion and also identify some who fall within the nonfamilial HCM group, which has important implications for the follow up care of family members [24]. When compared with a cohort of patients explicitly meeting diagnostic criteria for HCM, a heterogeneous HCM population specifically ascertained through referral for genetic testing may have a lower diagnostic yield, and a higher rate of clinically significant variants in genes outside those encoding the proteins of the sarcomere.

The aim of this study is to report on the diagnostic yield of genetic testing, outline the genes in which diagnostic variants were identified, by applying a systematic ACMG/AMP-compatible variant classification scheme and determine which clinical variables influence the likelihood of a diagnostic test result in a heterogeneous HCM cohort evaluated over a 5-year period.

## Methods

### Study patients

The cohort in this study included 1376 patients having been identified as having a suspected diagnosis of HCM by their ordering provider in their laboratory requisiton. HCM diagnostic criteria was not utilized to include or exclude patients. Complete clinical data was not available to verify the diagnoses. Only patients for whom panel testing was ordered were included. The patients were presumed to be affected and unrelated. Demographic, clinical and diagnosis information including age, sex, family history, documented arrhythmias, type of medical device and patient outcomes was obtained from requisitions completed by ordering providers. This work was reviewed by the Western Institutional Review Board (IRB) and received an exemption determination.

### Genetic testing 

Patients underwent testing as ordered by their healthcare provider for either an HCM panel (16, 19 or 38 genes), a broad cardiomyopathy panel (72, 101, 103, 134 or 155 genes) or a broad cardiology panel (133, 165 or 184 genes associated with arrhythmias/cardiomyopathies) (Additional file 1). All of the genes on the HCM panel are included in the broad cardiomyopathy and broad cardiology panels. A total of 1133 (82.3%) cases were analyzed using the oligonucleotide-selective sequencing (OS-Seq™) (25) NGS method on the NextSeq sequencing system (Illumina). The remaining patients (17.7%) were analyzed using an in-house tailored Integrated DNA Technologies (IDT) based whole exome sequencing platform run on the NovaSeq sequencing system (Illumina). In the analysis, mean sequencing depth was > 150× and > 99% of target nucleotides were covered with > 20× sequencing depth for all assays. The target nucleotides include all protein coding exons of the genes on the panels, as well as 20 base pairs (bp) inside each intron/exon boundary. Later versions of the panels were customized by adding oligonucleotides targeting deep intronic variants (≥ 20 bp from the intron/exon boundary) and non-coding variants [promoter region, 5′ or 3′ untranslated regions (UTR)] that have been reported as disease causing in association with cardiomyopathy and arrhythmia (Additional file 1). The sequence variant analysis pipeline has been validated in a CLIA (Clinical Laboratory Improvement Amendments) and CAP (College of American Pathologists) accredited Blueprint Genetics diagnostic laboratory. Bi-directional Sanger sequencing was used to confirm likely pathogenic (LP) and pathogenic (P) sequencing variants whenever stringent quality criteria for a true positive call were not met. The series of quality criteria utilized include a variant call quality score, variant genomic location, sequence content, and integrative genomics viewer visual analysis. This algorithm was established based on the outcome of an internal validation performed in the CLIA and CAP accredited Blueprint Genetics diagnostic laboratory.

### Copy number variant analysis

CNV analysis was performed bioinformatically from the NGS data using a bioinformatic pipeline; one component used for calling is CNVkit and another is an in-house developed proprietary technology. CNVs were confirmed using quantitative polymerase chain reaction (qPCR) technology. The CNV analysis pipeline has been validated in the CLIA and CAP accredited Blueprint Genetics diagnostic laboratory.

### Interpretation of test results

Variants were classified according to an adaptation of the ACMG/AMP guidelines [14] as outlined in the Blueprint Genetics website (https://blueprintgenetics.com/variant-classification/). Tests were considered diagnostic if the variants were classified as likely pathogenic (LP) or pathogenic (P). The Blueprint Genetics classification scheme is similar to the ACMG/AMP guidelines [14] in that multiple independent lines of evidence must be met (e.g. rare in population databases, predicted deleterious by in silico software, segregation with disease, de novo in a patient with no family history, damaging impact shown in well-established functional studies, etc.) to achieve a likely pathogenic or pathogenic classification, and several of these criteria are equivalent in both schemes. For example, LP missense variants are most often rare in population databases, predicted deleterious by in silico software tools, are reported in affected individuals/segregate in families and demonstrate a clear gene-phenotype association.

### Statistical analysis

Comparisons between groups were performed with either Fisher’s exact or Chi-Square test for categorical variables, as appropriate, and unpaired T-test for normally distributed continuous variables. P-values less than 0.05 were considered statistically significant.

## Results

Genetic testing was performed on 1376 cases of patients with a clinical suspicion of HCM. LP/P variants were identified in 369 cases, resulting in a diagnostic yield of 26.8%. Demographic and clinical variables are outlined in Table 1. LP/P variants were identified in 30.8% (151/491) of women and 24.7% (218/884) of men (*p* = 0.015). In one case (n = 1), the patient’s gender was not specified. A broad cardiomyopathy panel was ordered for 51.1% of cases (n = 703); an HCM specific panel was ordered for 45.9% (n = 632), and the remainder underwent a broad cardiology (cardiomyopathy and arrhythmia genes) panel (n = 41). The diagnostic yield was highest for the broad cardiology panel (31.7%, n = 13), but the overall yield was not significantly higher when compared to the HCM-specific panel (24.4%, n = 154, *p* = 0.29) or the broad cardiomyopathy panel (28.7%, n = 202, *p* = 0.68) (Table 2). Both sequencing and deletion/duplication analysis were completed in 40.3% of cases (n = 554); the remainder had sequence variant analysis alone.

**Table 1:** Patient demographic and clinical variables

	Number of cases	%
Age at diagnosis (n = 769)		
Infant (< 1 year)	35	4.6
Child (1–17 years)	72	9.4
Adult (≥ 18 years)	662	86.1
Average	769	44.7
Gender (n = 1376)		
Female	491	35.7
Male	884	64.2
Unknown/not provided	1	0.1
Positive family history (n = 1376)	421	30.6
Clinical findings		
Maximum wall thickness > 16 years (n = 849)		
≤ 12 mm	39	4.6
13–14 mm	45	5.3
15–20 mm	457	53.8
21–25 mm	228	26.9
≥ 26 mm	80	9.4
Hypertension (n = 1376)	158	11.5
Aortic stenosis (n = 1376)	8	0.6
AV block (n = 1376)	15	1.1
Ventricular tachycardia (n = 1376)	115	8.4
Resuscitated cardiac arrest (n = 1376)	42	3.1
ICD (n = 1376)	108	7.8
Heart transplantation (n = 1376)	18	1.3

*AV Block* for atrioventricular block, *ICD* for implantable cardioverter-defibrillator

**Table 2:** Overall diagnostic yield by panel type

Panel type	Number of genes/panel	Number of panels ordered (% of total)	Number of diagnostic tests	Number of diagnostic variants	Diagnostic yield (%)
Broad cardiomyopathy	72–155	703 (51%)	202	205	28.7
Broad cardiology	133–184	41 (3%)	13	13	31.7
HCM	16–38	632 (46%)	154	155	24.4
Total		1376	369	373	26.8

Overall diagnostic yield presented by panel type. Percentages in column “number of panels ordered” represent the proportion of all panels ordered (n = 1376)

*HCM* for hypertrophic cardiomyopathy

### Variant profile

Altogether, 373 P/LP variants were identified in 31 unique genes (Additional file 2). Most (85.0%, n = 317) of the diagnostic variants were in genes encoding sarcomere proteins, followed by the RASopathy genes (4.3%, n = 16), non-sarcomere HCM genes (4.3%, n = 16), metabolic/storage disease genes (3.5%, n = 13) and other cardiomyopathy genes (1.3%, n = 5). The remainder were in genes associated with myopathy, arrhythmias/channelopathies and neurofibromatosis (n = 6).

The HCM-specific panel yielded the highest proportion of sarcomere variants (92.3%, n = 143) compared to the broad cardiomyopathy (80.5%, n = 165) and broad cardiology (69.2%, n = 9) panels. The broad cardiology panel yielded the highest proportion of diagnostic variants in non-sarcomere HCM genes (15.4%, n = 2) compared to the broad cardiomyopathy (5.9% n = 12) and HCM-specific panel (1.3% n = 2).

**Fig. 1:**
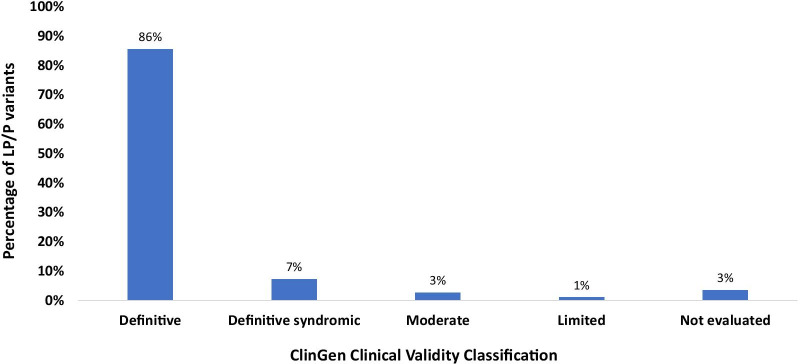
Distribution of LP/P gene by ClinGen HCM Gene Disease Association. Bar chart demonstrates the distribution of LP/P gene by ClinGen HCM Gene Disease Association. The Y axis indicates percentage of all diagnostic variants (n = 373) and the X axis indicates the ClinGen disease association. *LP* for likely pathogenic, *P* for pathogenic and *HCM* for hypertrophic cardiomyopathy. Genes included in sections: Definitive: *ACTC1, MYH7, MYL2, MYL3, TNNI3, TNNT2, TPM1, MYBPC3, PLN*; Definitive syndromic: *DES, FHL1, FLNC, GLA, LAMP2, PRKAG2, RAF1, TTR*; Moderate: *JPH2*; Limited: *TTN, RYR2*; Not evaluated: *CPT2, DSG2, HRAS, JUP, LMNA, MAP2K1, NF1, SCN5A, SHOC2, TRPM4*

According to the ClinGen gene validity evaluation [18], 95.4% (n = 356) of LP/P variants were identified in genes that are definitively or moderately associated with HCM and LVH, an overall yield of 25.8% for the cohort. The remaining variants (n = 17) were in genes that have not been evaluated or have limited evidence of an association with HCM/LVH (Fig. 1).

**Fig. 2:**
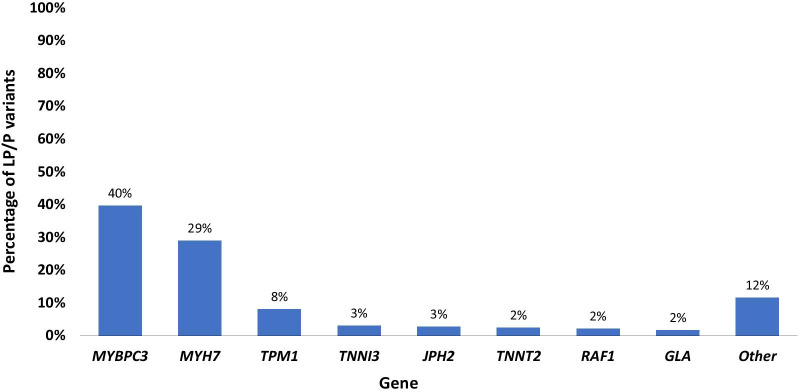
Distribution of Diagnostic Variants by Gene. Bar chart demonstrates the distribution of diagnostic variants by gene as a percentage of total variants (n = 373). The Y axis indicates the percentage of all diagnostic variants (rounded to the nearest whole percent) and the X axis indicates the gene. Section titled “*Other*” includes genes that had five variants or less detected. Genes with ≤ 5 variants detected: *MYL2* (n = 5), *PTPN11* (n = 4), *DES* (n = 3), *TTN* (n = 3), *LMNA* (n = 2), *FHL1* (n = 2), *FLNC* (n = 2), *HRAS* (n = 2), *LAMP2* (n = 2), *MYL3* (n = 2), *PRKAG2* (n = 2), *TTR* (n = 2), *DSG2* (n = 2), *CPT2* (n = 1), *MAP2K1* (n = 1), *SHOC2* (n = 1), *ACTC1* (n = 1), *PLN* (n = 1), *TRPM4* (n = 1), *JUP* (n = 1), *NF1* (n = 1), *RYR2* (n = 1), *SCN5A* (n = 1)

LP/P variants were seen most often in *MYBPC3* (39.7%, n = 148) *MYH7* (29.0%, n = 108) followed by *TPM1* (8.0%, n = 30), *TNNI3* (2.9%, n = 11), *JPH2* (2.7%, n = 10), *TNNT2* (2.4%, n = 9), *RAF1* (2.1%, n = 8) and *GLA* (1.6%, n = 6). The remaining 23 genes each had five variants or less (Fig. 2).

Loss of function variants in *MYBPC3* and missense variants in *MYH7* were the most common LP/P variants seen. A single CNV was identified in exon 3 of the *RYR2* gene.

Table 3 illustrates the breakdown of the most commonly implicated genes and variant types.

**Table 3:** Variant type and classification in genes (> 5 LP/P variants)

Gene	Pathogenic	Likely Pathogenic	LoF	Splice	Missense	Inframe deletion
*MYBPC3*	101 (27.1%)	47 (12.6%)	81 (21.7%)	39 (10.5%)	23 (6.2%)	5 (1.3%)
*MYH7*	75 (20.1%)	33 (8.8%)	0	0	106 (28.4%)	2 (0.5%)
*TPM1*	21 (5.6%)	9 (2.4%)	0	0	30 (8.0%)	0
*TNNI3*	7 (1.9%)	4 (1.1%)	0	0	11 (2.9%)	0
*JPH2*	10 (2.7%)	0	0	0	10 (2.7%)	0
*TNNT2*	7 (1.9%)	2 (0.5%)	0	0	7 (1.9%)	2 (0.5%)
*RAF1*	3 (0.8%)	5 (1.3%)	0	0	8 (2.1%)	0
*GLA*	3 (0.8%)	3 (0.8%)	2 (0.5%)	0	4 (1.1%)	0

Variant types and classification of likely pathogenic and pathogenic variants in genes in which more than five variants were detected. Percentages represent the proportion of total variants identified (n = 373)

*LP* for likely pathogenic, *P* for pathogenic, *LoF* for Loss of function, *Splice* for consensus splice site variant or other variant with known or suspected effect on splicing. A total of five splice variants were at a position greater than ± 10 bp from the intron/exon boundary

### RASopathy findings

A total of 959 tests included at least two RASopathy genes. A LP/P variant was found in 1.7% (n = 16) of these, representing 4.3% of all LP/P variants. The majority (81.3%, n = 13) of all diagnostic variants in RASopathy variants were identified on the broad cardiomyopathy panel; the remainder were from the HCM-specific panel. In 15/16 cases, the RASopathy gene variant was the only LP/P variant identified, in one case two LP variants were seen; (*RAF1* and *TNNT2*). Variants were seen most commonly in the *RAF1* gene (n = 8), followed by *PTPN11* (n = 4), *HRAS* (n = 2), *MAP2K1* (n = 1) and *SHOC2* (n = 1). The mean age at diagnosis in the RASopathy group was 19.1 years, which is significantly lower than seen in patients without a RASopathy variant (33.8 years, *p* = 0.002). Syndromic features were reported by the clinician for 31.3% of patients (n = 5), all but one of whom had a RASopathy variant as their only genetic finding. All patients were heterozygous for their identified variant; no patients showed evidence of mosaicism within the limits of what is detectable by the assay.

### Other findings

A total of 0.9% (n = 13) of all patients had LP/P variants in genes associated with a metabolic or storage disease. Just over half of these were identified from the HCM-specific panel (53.8%, n = 7) and the remainder from the broad cardiomyopathy panel (n = 6). Most were in the *GLA* gene (n = 6), followed by 2 patients with variants in *TTR, PRKAG2* and *LAMP2* respectively and finally one with *CPT2*. A total of 0.6% of cases (n = 8) had LP/P variants in genes associated with non-HCM cardiomyopathy (two in *LMNA,* and *DSG2,* one in *JUP*) or arrhythmias (one in *SCN5A, RYR2, TRPM4* respectively). One third of channelopathy/arrhythmia genes were identified on the broad cardiomyopathy panel. The remainder of the channelopathy/arrhythmia variants were identified on the broad cardiology panel. Diagnostic variants in syndromic (n = 1), other cardiomyopathy (n = 5) and myopathy genes (n = 1) were only identified from the broad cardiomyopathy panel. Of all cases, 0.3%  (n = 4) had two or more LP/P variants.

### Clinical variables and diagnostic yield

**Fig. 3:**
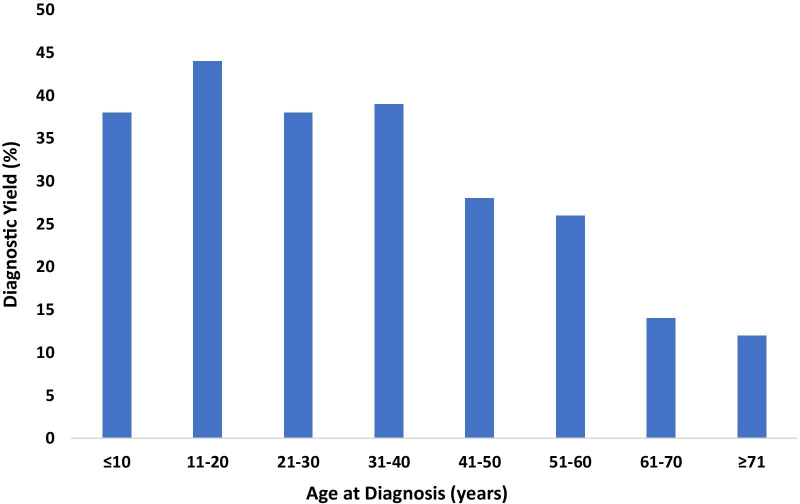
Diagnostic Yield (LP/P variants) by Age at Diagnosis (Where Reported). The Y axis indicates percentage of diagnostic yield, and the X axis indicates age at diagnosis

Age at diagnosis had a significant effect on the likelihood of identifying a LP or P variant. The average age at diagnosis was 37.2 years in those with a diagnostic finding (reported in 216/369) and 47.6 years for those with no diagnostic findings (reported in 553/1007), *p* = 6.4 × 10^–10^. The diagnostic yield was highest for patients who were 11–20 years of age at the time of diagnosis, (45.8%, reported in 33/72) and lowest for patients who were 61–70 years of age at the time of diagnosis (17.5%, reported in 21/120) (Fig. 3). In considering only genes in which 5 or more LP/P variants were found, the age at diagnosis was the lowest (26.5 years) in patients who had a missense variant in *TNNT2* and the highest (46.3 years) in those with a missense variant in *GLA*.

A positive family history was significantly associated with the likelihood of identifying a LP/P variant. LP/P variants were significantly more common in patients with a family history (37.1%; 156/421) than in those without a family history (22.3%; 213/955) (*p* < 0.0001).

**Fig. 4:**
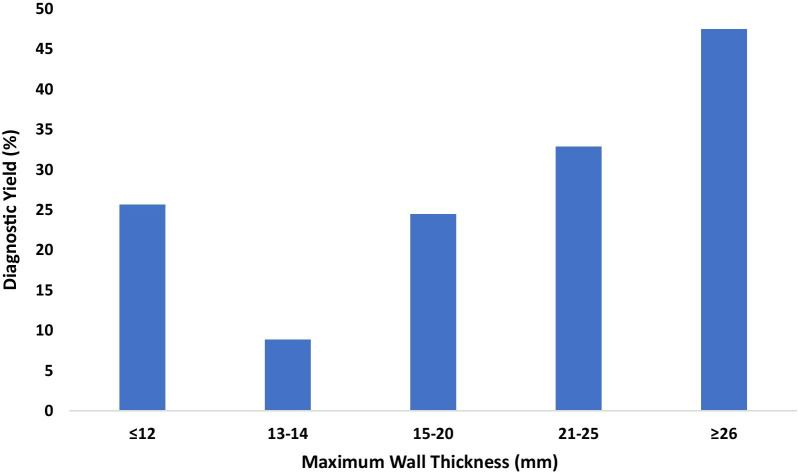
Diagnostic Yield (LP and P variants) by Maximum Wall Thickness (mm). The Y axis indicates percentage of diagnostic yield, and the X axis indicates the maximum wall thickness in millimeters. *mm* millimeter

Higher maximum wall thickness (MWT) increased the likelihood of identifying a LP or P variant. MWT was higher (20.7 ± 4.8 mm and 19.0 ± 4.2 mm, *p* = 2.1 × 10^–7^) in patients over 16 years old with a diagnostic finding (reported in 239/849) compared to those patients with no molecular diagnosis (reported in 610/849). Patients older than 16 years of age with a LP/P variant in a ClinGen definitive HCM gene had a MWT that was similar to patients older than 16 years of age with LP/P variants in ClinGen moderate, limited evidence/not evaluated HCM genes (20.9 ± 4.8 and 19.6 ± 5.0, *p* = 0.22). Where MWT information was available, the diagnostic yield was the highest in patients with a wall thickness of ≥ 26 mm (47.5%, 38/80) and the lowest in patients with a maximum wall thickness of 13–14 mm (8.9%, 4/45) (Fig. 4). In considering only genes in which 5 or more variants were identified, the greatest MWT was seen in patients with a splice site LP/P variant in *MYBPC3* (22.8 mm) and smallest in those with a LP/P variant in *TNNT2* (14.5 mm)*.*

The absence of hypertension significantly affected the likelihood of identifying a LP/P variant. Of all patients without hypertension, 28.4% (346/1218) had a LP/P variant identified. Of all patients with hypertension, 13.9% (22/158) had a LP/P variant identified (*p* = 0.0002).

Patients with an implantable cardioverter-defibrillator (ICD) were more likely to have a LP/P variant identified (41.7%, 45/108) compared to those without an ICD (25.6%, 324/1268) (*p* = 0.0004). Clinical variables not significantly associated with the likelihood of identifying a LP/P variant were atrioventricular (AV) block, aortic stenosis, heart transplantation, ventricular tachycardia, cardiac arrest, pacemaker or whether previous genetic testing had been performed.

## Discussion

Genetic testing for HCM is more accessible than ever and is supported by multiple international societies [1, 23]. Describing the genetic findings and diagnostic yield in a large, heterogeneous, cohort of patients referred for genetic testing further informs clinicians about the likelihood of obtaining a diagnostic result and the breadth of genes in which these variants may be found as to further tailor counseling, surveillance and management strategies.

A retrospective review of 1376 probands with a suspected diagnosis of HCM as indicated by the ordering provider and referred for genetic testing via NGS technology over a 5-year period identified a diagnostic finding in 26.8% (n = 369/1376) of cases. This yield is lower than previously reported in other studies [31.5%; 917/2912 (9), 32.3%; 1998/6179 (26), 33.3%; 129/387 (11), 33.2%; 127/382 (12), 46.7%; 491/1198 (13), 54.3%; 38/70 (10)]. Our cohort of HCM patients is one of the most heterogeneous reported to date, as this study only included cases where the clinician specifically indicated a suspicion of HCM; no supporting diagnostic criteria were required for inclusion and no clinical data excluded patients. Although the inclusion criteria utilized here aren’t as stringent as those used for some clinical cohorts, they allow for the reporting of the diagnostic yield in all patients with suspected HCM referred for genetic testing. The lower yield reported in this study could be explained by the inclusion of patients with a low suspicion of HCM, including those with an alternate explanation for LVH such as systemic findings and/or a syndrome. Other comparable cohorts such as the cohort of 2,912 HCM patients reported by Alfares et al*.* excluded individuals with LVH explained by a clinical syndrome and unaffected individuals with only a family history [9]. In our study, nearly 10% of patients who were > 16 years of age at the time of testing were reported to have a MWT of 14 mm or less, raising the possibility that these patients may have an alternative diagnosis. It should also be noted that the high diagnostic yield reported in some of these earlier studies may be explained by the inclusion of variants in genes that have now been shown to have limited or no evidence of being associated with HCM [18], or by including all suspicious variants in the total yield. For example, Rubattu et al*.* [10] reported a high diagnostic yield (54.3%; 38/70) but these included some debatable findings; for example, three ‘causative’ variants in *MYH6* and *CAV3*. According to ClinGen or the Genomic England PanelApp, neither of these two genes are considered to be associated with HCM at gene level or without overt syndromic features [18, 27]. The study by Mazzarotto et al*.* [13] includes P, LP and variants of uncertain significance (VUS) in their total diagnostic yield. Finally, earlier versions of the Blueprint Genetics HCM panel (see Additional file 1: Table 1) did not include the now well-established HCM/left ventricular hypertrophy genes *PLN* and *PTPN11*, which might also have an impact on overall yield.

Notably, 4.6% of all LP/P variants were in genes deemed to have limited evidence, or not evaluated in association with HCM [18]. Also, 2.4% of all LP/P variants, representing 0.7% of all cases, were in genes that are not typically included on an HCM panel (*LMNA, JUP, DSG2, SCN5A, RYR2, TRPM4, NF1*). These genes are associated with other cardiomyopathies, arrhythmias or syndromes, which have different treatment or management strategies and so are of the utmost importance to identify. Sufficient clinical data was not available to verify the accuracy of the HCM diagnoses in patients with a LP/P variant in *LMNA, JUP, DSG2, SCN5A, RYR2, TRPM4* or *NF1*. Diagnostic variants in the broad cardiomyopathy panel were the most varied and were distributed across all gene categories (sarcomere, RASopathy, non-sarcomere HCM, metabolic/storage, other cardiomyopathy, channelopathy/arrhythmia and syndromic/myopathy). This would be expected as the broad cardiomyopathy and broad cardiology panels include a number of other cardiomyopathy, channelopathy, channelopathy/arrhythmia, syndromic/myopathy genes that are not included on the HCM-specific panel. Further, patients offered a broad cardiomyopathy panel may have a lower index of suspicion for HCM or an unclear cardiomyopathy presentation. Unfortunately, there is limited clinical information available on individual patients, and so it is difficult to draw conclusions about specific features that prompted choosing a broader cardiomyopathy/arrhythmia panel over an HCM-specific one. Finally, the MWT in patients over 16 years of age at the time of testing was similar in patients with LP/P variants in definitive, strong, moderate, limited or not evaluated ClinGen HCM genes [18]. Higher MWT in patients with variants in non-HCM related genes could be explained by other factors that were not described in the requisition or due to possible discrepancies in imaging measurements.

The prevalence of RASopathy LP/P variants in patients whose panel included at least two or more RASopathy genes was more than double what has previously been published in a similar HCM cohort (1.7% vs. 0.7%) [20]. Our reported proportion may be an underestimate of the true prevalence of RASopathy findings, as earlier versions of the HCM and cardiomyopathy panels utilized in this study did not include the most common of the RASopathy genes associated with left ventricular hypertrophy (*PTPN11, RAF1, RIT1*). Despite small numbers, it is worth noting that patients harboring RASopathy LP/P variants were significantly younger than those with non-RASopathy LP/P variants. A systematic evaluation of syndromic features in these patients was not possible as limited clinical data was available. Further studies are needed to better understand the phenotypic spectrum of these patients presenting with apparently isolated LVH. Our work supports the inclusion of RASopathy genes on HCM specific panels.

This study reports a higher proportion of *JPH2* diagnostic variants (2.7%) than has been previously reported, all of which were the c.482C > A; p. (Thr161Lys) variant. A recent publication by our group describes nine Finnish HCM families with this variant and no other LP/P variant which would cause HCM [28], suggesting that this could be a founder variant in Finland. Segregation with disease was seen in 6 of these families [28]. This *JPH2* missense variant is also absent in population databases (gnomAD) and predicted to be deleterious by the majority of in silico tools used. The laboratory where testing was performed is located in Finland and is used by Finnish providers, thus explaining the higher proportion of patients with this one specific variant, in a gene that is not commonly associated with HCM.

We identified a small proportion (0.3%)of patients in this cohort with multiple LP/P variants, which is comparable to what has recently been reported (0%) in HCM using strict ACMG/AMP classification criteria [12]. This further supports the notion that HCM patients rarely have more than one LP/P variant.

None of the deep intronic (≥ 20bps from the intron/exon boundary) or non-coding (located in the promoter or 5′ and 3′ UTR) variants included in later versions of the panels (Additional file 1) were identified in this patient cohort. Only 1.3% of all LP/P variants identified (5/373) were splice variants at positions greater than ± 10 bp, which is what is covered by most NGS panels. These two variants were *MYBPC3* c.1224-19G > A (n = 1) and *MYBPC3* c.1227-13G > A (n = 4). Including ± 20 bp from the intron/exon boundary in the target region of this analysis increased the diagnostic yield from 26.5% (364/1373) to 26.8% (369/1373). The deep intronic variants recently identified by Bagnall et al*.* [29] such as *MYBPC3* c.1090 + 453C > T, *MYBPC3* c.1091-575A > C, *MYBPC3* c.1224-52G > A which increased their diagnostic yield by 8.7%, were not included in the analysis of any of our patients. The inclusion of non-coding regions brings a challenge to the interpretation of variants when accompanying RNA studies are not available. Therefore, this study included only deep intronic variants (≥ 20 bps from the intron/exon boundary) or non-coding variants (such as those in the promoter, 5′ or 3′ UTRs) listed in Additional file 1 that have a previously established disease association or known splice defect in their target region at the time the analysis was performed. More extensive analysis of deep intronic and non-coding variants in this cohort is needed to determine whether they may have a greater impact on diagnostic yield.

Despite the above differences, several aspects of our findings are comparable to, and support what has previously been published in HCM cohorts from all genetic testing eras. The use of a broader, larger genetic testing panel did not lead to a significant increase in diagnostic yield, which has been demonstrated by others [12, 17] even when compared to whole genome sequencing [30]. In this study, LP/P variants were most commonly found in genes encoding the proteins of the sarcomere, the majority being in *MYBPC3* and *MYH7,* as has been previously demonstrated [5, 7, 9, 31]*.* We identified a single CNV in this study (0.1% of all cases) in a gene not associated with HCM (*RYR2*). This represents a lower proportion than what has previously been reported using NGS technologies (1.3% (11) and 0.6% (15)). Our data further demonstrates that CNVs are rare in HCM patients. However, it is worth noting that less than half of the cases (40.3%) in this study underwent both sequencing and deletion/duplication analysis.

Finally, a younger age of onset, a greater MWT, the absence of hypertension and a family history of HCM were all individually, significantly associated with a greater likelihood of identifying a LP/P variant. These are well known clinical variables that have been integrated into both the Mayo [8] and Toronto [22] scores for predicting the yield of genetic testing in HCM patients. One important limitation to our family history analysis is that if the family history section in the requisition was left incomplete, it was assumed that the family history was negative. The diagnostic yield in patients with a MWT of 13–14 mm was lower than that of those with a MWT ≤ 12 mm. Without additional clinical details, this finding is difficult to explain. We found that the presence of an ICD was associated with a higher likelihood of identifying a LP/P variant, which has been shown previously [6]. Because of the limited information provided in the test requisitions, we were not specifically able to evaluate whether patients with negative genetic test results fit into a non-familial HCM group, which is defined by Ingles et al*.* by HCM index patients with a low Toronto score [22], a negative family history including at least two adult children, and no sarcomere variants identified [24]. Although we were not able to establish a proportion of true non-familial HCM patients in our cohort, it is of value for providers to appreciate the clinical variables associated with a negative result, particularly in a heterogeneous cohort that likely includes patients with a lower index of suspicion for HCM.

## Conclusions

The diagnostic yield in this heterogeneous cohort of patients is lower than what has previously been reported in other published cohorts, likely due to differences in patient cohorts, study inclusion criteria, improvements in variant classifications and HCM gene curation efforts. The majority of LP/P variants were in genes that are definitively associated with HCM [18]. An important proportion of patients referred for HCM genetic testing have a LP/P variant in a RASopathy gene. An evaluation for syndromic features is warranted, as the phenotypic spectrum of the RASopathy syndromes continues to expand. A comprehensive inclusion of RASopathy genes on HCM panels may increase the diagnostic yield, and the findings in these genes have important implications for patients given the need for extra-cardiac management. Offering a broader panel may be of value in some patients as, despite a clinical suspicion of HCM, their presentation may actually be explained by a different diagnosis. Further work is needed to understand how to increase the diagnostic yield of genetic testing in patients with HCM. Thus far, the analysis of deep intronic/non-coding variants has been shown to provide the most substantial increase in diagnostic yield [29], more so than new gene discovery at this time.

## Supplementary Information

**Additional file 1**. Genetic Testing Panels Ordered for Study Patients.

**Additional file 2**. Likely Pathogenic and Pathogenic Variants Identified in the Study Patients.

## Data Availability

The datasets used and/or analysed during the current study are available as Additional files 1, 2: Tables 1 and 2. Additional data is available from the corresponding author on reasonable request.

## Abbreviations

ACMG: American College of Medical Genetics and Genomics
AMP: Association for Molecular Pathology
AV block: Atrioventricular block
Bp: Base pairs
CAP: College of American Pathologists
CLIA: Clinical Laboratory Improvement Amendments
CNV: Copy Number Variant
HCM: Hypertrophic cardiomyopathy
ICD: Implantable cardioverter-defibrillator
IDT: Integrated DNA Technologies
LoF: Loss of function
LP: Likely pathogenic
LV: Left ventricle
LVH: Left ventricular hypertrophy
MLVWT: Maximum left ventricular wall thickness
Mm: Millimeter
MWT: Maximum wall thickness
NGS: Next generation sequencing
OS-Seq™: Oligonucleotide-selective sequencing
P: Pathogenic
qPCR: Quantitative polymerase chain reaction
UTR: Untranslated region
VUS: Variants of Uncertain Significance
